# Response to Stutz et al. re: “Management of Suspected Bladder Injury and Capsular Perforation After Holmium Laser Enucleation of the Prostate”

**DOI:** 10.1089/cren.2018.0089

**Published:** 2018-12-20

**Authors:** Aye Lwin, David Tzou, Joel Funk

**Affiliations:** Division of Urology, Arizona Health Sciences Center, University of Arizona, Tucson, Arizona.

We appreciate the review provided by Stutz et al. in response to our case report on the management of capsular perforation during holmium laser enucleation of the prostate (HoLEP). There are several valid points made by the authors with regard to the safety, efficacy, and durability of HoLEP for patients with outlet obstruction secondary to prostatic enlargement. The letter gave a concise overview of the proper safety standards to prevent bladder injury during morcellation for patients undergoing this operation.^1^

The purpose of our case report was not to emphasize prevention and management of a bladder injury/perforation, which has been well documented previously.^2,3^ Instead, it was to share that in our experience of >800 cases at our institution since 2009, these 2 cases were memorable as they demonstrate how prostatic capsular perforation can resemble the intraoperative signs of a bladder injury. It should be reiterated that, in both cases, neither patient actually suffered a bladder injury from morcellation. Yet, the physical examination findings of a distended abdomen, paired with changes in peak airway pressures or a mismatch in irrigant inflow and outflow resembled that of a bladder injury. As the case report highlights, the proper management of this clinical situation is vastly different from that of exploring for a suspected bladder injury, as capsular perforation can be managed conservatively with Lasix and bladder decompression.^4^

The steep learning curve for HoLEP has been well documented and experienced HoLEP practitioners will recognize that visualization during morcellation can be impacted by multiple factors, including hemostasis, degree of bladder distention, prostatic size, prior indwelling catheter, and the concomitant administration of anticoagulants. If safe technique during morcellation is adhered to with low suspicion of bladder perforation, the etiology of the physical examination findings can be attributed to a capsular perforation. Urologists performing HoLEP should be aware of all the potential complications of HoLEP, and the nuances in their presentation, work-up, and management.

## Abbreviation Used

HoLEP: holmium laser enucleation of the prostate
